# A Hybrid Framework for Understanding and Predicting Human Reaching Motions

**DOI:** 10.3389/frobt.2018.00027

**Published:** 2018-03-27

**Authors:** Ozgur S. Oguz, Zhehua Zhou, Dirk Wollherr

**Affiliations:** Department of Electrical and Computer Engineering (EI), Technical University of Munich (TUM), Munich, Germany

**Keywords:** inverse optimal control, human motion modeling, reaching motion prediction, human-in-the-loop control, human–robot collaboration, probabilistic movement primitives

## Abstract

Robots collaborating naturally with a human partner in a confined workspace need to understand and predict human motions. For understanding, a model-based approach is required as the human motor control system relies on the biomechanical properties to control and execute actions. The model-based control models explain human motions descriptively, which in turn enables predicting and analyzing human movement behaviors. In motor control, reaching motions are framed as an optimization problem. However, different optimality criteria predict disparate motion behavior. Therefore, the inverse problem—finding the optimality criterion from a given arm motion trajectory—is not unique. This paper implements an inverse optimal control (IOC) approach to determine the combination of cost functions that governs a motion execution. The results indicate that reaching motions depend on a trade-off between kinematics and dynamics related cost functions. However, the computational efficiency is not sufficient for online prediction to be utilized for HRI. In order to predict human reaching motions with high efficiency and accuracy, we combine the IOC approach with a probabilistic movement primitives formulation. This hybrid model allows an online-capable prediction while taking into account motor variability and the interpersonal differences. The proposed framework affords a descriptive and a generative model of human reaching motions which can be effectively utilized online for human-in-the-loop robot control and task execution.

## 1 Introduction

As robots become more present in our social lives, the necessity for interaction and collaboration between humans and robots is becoming more apparent. Although there are several major facets of providing robots with such capability, e.g., motion planning or decision-making, the human aspect has to be prioritized and integrated into robot interaction skills. Requirements for such a human-in-the-loop formulation is twofold: describe (*understand*) how human motions are controlled and generate (*predict*) human-like motions. A descriptive model helps us understand how the biomechanical properties are used by the central nervous system (CNS) for controlling human body to execute a vast collection of motor behaviors. Such an understanding is useful for a multitude of problems, e.g., motor performance evaluation for detecting disabilities due to neural disorders by comparing control models of patients and healthy subjects (Manto et al., 2012); sports performance evaluation by analyzing the identified control models of athletes (Yarrow et al., 2009); detection of deviations of personal motion behaviors w.r.t. the previously identified motor control models, e.g., due to exhaustion (Shadmehr et al., 2010). Specifically, for human–robot interaction (HRI), the robot can plan its motions in a way to allow the human partner to rely more on energy-efficient control models. In addition, person-specific control models enable the robot to detect the underlying cause of behavioral anomalies for providing better assistance and safety.

A generative model allows estimating human-like motion trajectories. In this work, the focus is using such models to *predict* human motions, rather than transferring them to robots to generate human-like movement behaviors. For close dyadic collaboration, where the partners share a workspace with the possibility of overlapping motions, they should be able to predict each other’s intent and the required motion that can support this intention. Considering how swiftly two humans work together in a confined workspace, the challenges for a human–robot team become obvious; the robot has to take into account human partner’s intention and movement in order to control its own motion for achieving effective cooperative task executions. In essence, early prediction of the human motion allows an immediate initiation of the replanning process and an early adaptation of the robot motion (Dinh et al., 2015; Gabler et al., 2017; Oguz et al., 2017). Therefore, the ability of understanding and predicting human motions effectively is the key to achieving swift close human–robot collaboration.

**Figure 1:**
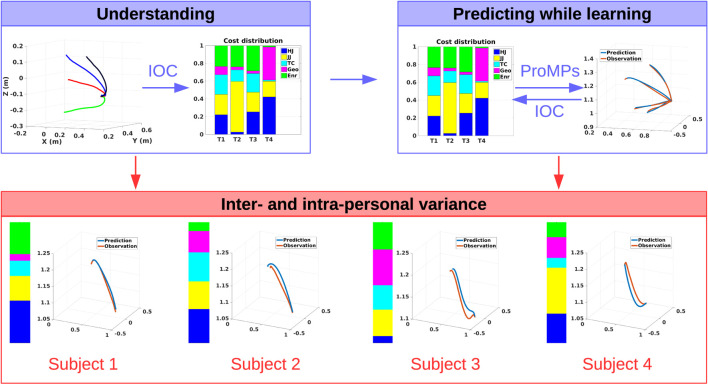
The overall framework, where the focus is twofold: understanding (upper left) and prediction (upper right) of human motion behaviors. For understanding, biomechanically inspired cost function distributions are learned from demonstrations by model-based inverse optimal control; and for online prediction, data-driven probabilistic movement primitives are used. The two approaches are interconnected to each other in order to account for the inter-and intra-personal movement behavior variations in terms of both motion trajectories and also the cost distributions.

The focus in this work is twofold. First, descriptive models of human reaching motions are investigated and experimentally evaluated. Second, a hybrid framework is proposed, which combines those descriptive models with a data-driven probabilistic approach and realizes online-capable human motion prediction (Figure 1). Such a framework not only enables effective robot control for human-in-the-loop scenarios but also they can be directly used for controlling the robot.

Currently, there is no commonly accepted model that explains how the human CNS controls human motions and the latent biomechanical properties of the human motion are not fully understood. Knowing the underlying principles of human motion execution is essential for reproducing human-like motion behaviors accurately in a given setting. However, not every single person exhibits the same motion patterns. These differences might be due to their learning experiences and physiological differences (Rosenbaum, 2009). Moreover, even the motion behaviors of the same person show variations due to motor noise (Todorov and Jordan, 2002). Considering all those intricacies, finding motion behavior models, even for simple reaching tasks, poses challenging research questions.

As the observations of the human motions’ behavioral aspect suggest an appealing modeling problem, the human body as a biomechanical system introduces challenges in terms of formulating methods for finding those models. Motor control redundancy and the non-linear characteristic of the human arm as a dynamical system are the most important problems to tackle. A common feature of motor control is that the task requirements can be met by infinitely many diverse movements. Thus, stating only the boundary conditions of the motion for given dynamics leads to an ill-defined problem. The ambiguity caused by this problem can be resolved if an optimality principle is applied. Accordingly, the basis of many scientific theories on human motor control is formed by optimality principles (Engelbrecht, 2001). A large number of models of open-loop motor control exist and each model claims to describe human motion, but several models are incompatible with others (Todorov, 2004). The characteristics of the human arm movements and the human as an organism define the starting point for the derivation of a cost function. Many cost functions have been proposed to model human reaching motions, however, all of those methods are only verified for specific settings, mostly in 2D (Flash and Hogan, 1985; Uno et al., 1989; Harris and Wolpert, 1998). Hence, their generalization capability to a wider range of human reaching motion behavior in 3D space is unclear. Moreover, as some recent studies suggest, humans might be optimizing two classes of cost functions, one for kinematics and the other for dynamics (Berret et al., 2011; Albrecht et al., 2012). However, finding the contribution of such multiple cost functions is also not trivial as it is a non-linear optimization problem.

Building on the results of prior research studies and their insights, we hypothesize humans utilize multiple models, rather than a single one, to control their motions. Since kinematics is essential for producing smooth motions, and the human arm is a dynamical system, it is reasonable to consider kinematics and dynamics related costs in combination. Hence, we identify possible costs from literature to account for both aspects. In order to find the contribution of each model for the realization of human motion behaviors, we frame such an inverse optimal control (IOC) problem as a bi-level optimization formulation. However, this formulation treats the human motion generation as a deterministic problem. In essence, it is only suitable for modeling average behavior over a group of humans. In order to afford both intra- and interpersonal motion variability, we propose a hybrid framework by extending the IOC formulation with a data-driven probabilistic method. Specifically, by utilizing probabilistic movement primitives (ProMPs), our framework allows for integrating person-specific variations into the IOC-based average motion behavior models during online interaction. Therefore, we can learn a distribution of motion behavior per person, and rollout predictive trajectories from this distribution online, while updating at the same time the multiple model representation to describe the person-specific cost optimization behavior.

**Figure 2:**
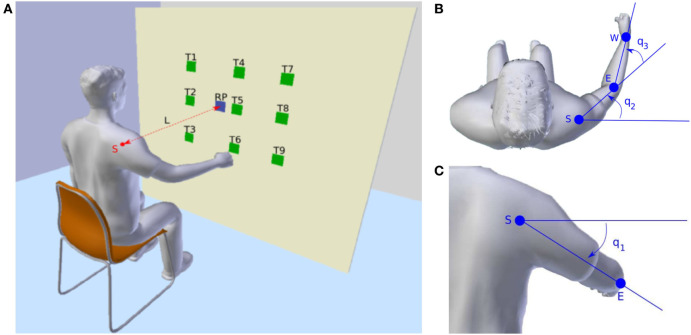
Experimental setup. **(A)** Overview of the experimental setup. T1 to T9 denote the nine target areas. RP means the reference point used to adjust the sitting position of the subject. S is the center of the shoulder joint and L is the distance between S and RP which is defined as 80% of the subject’s arm length. **(B)** Top view of the subject. S, E, and W are the shoulder joint, the elbow joint, and the wrist joint, respectively. As in the arm model defined, *q*_2_ and *q*_3_ are the yaw rotation of the shoulder joint and the pitch rotation of the elbow joint. **(C)** Back view of the subject. *q*_1_ is the pitch rotation of the shoulder joint.

We conducted a comprehensive experiment in 3D (Figure 2) that covers significantly more cases than prior studies (Albrecht et al., 2011). This extended experiment provides us with critical insights on the interplay between the parameters of the reaching tasks and the contribution of kinematics and dynamics related models. We identify a trade-off between those models with respect to the initial and final joint angle configurations. With the proposed hybrid framework, we are able to determine personal preferences as well as the motor variability per person. It also enables accurate and computationally efficient online prediction of human motion behaviors, which can be integrated into any human–robot collaboration scenario.

In this work, we focus on building descriptive as well as generative models for human motion behavior. By utilizing such models, we aim for efficient and accurate prediction of human motions during human–robot collaboration to realize a natural interaction between partners. To that end, the main contributions of this paper are:

– We propose a hybrid framework, consisting of a model-based approach and a data-driven probabilistic method, for predicting human motions.
– We identify a trade-off between kinematics and dynamics related costs depending on the reaching task.
– Our hybrid framework takes into account interpersonal differences and person-specific motor variability during online observations.

## 2 Related Work

Many experimental studies have revealed that arm motions exhibit invariant parameters which do not significantly change with movement speed, load, or direction (Soechting and Lacquaniti, 1981; Lacquaniti and Soechting, 1982; Papaxanthis et al., 2003). For motor control, these parameters are utilized to describe point-to-point reaching motions (Soechting and Flanders, 1991). It is assumed that the CNS follows some specific principles when planning the motions (Engelbrecht, 2001). Therefore, optimal control theory becomes the central mathematical formulation to model, describe, and understand motor control by the CNS (Bertsekas et al., 1995; Todorov, 2004), as it emphasizes the optimality of biological movements by minimizing some performance criteria. In literature, several optimal control models have been proposed to describe the point-to-point arm movements, e.g., the minimum hand jerk (Flash and Hogan, 1985), the minimum torque change (Uno et al., 1989), and the minimum variance (Harris and Wolpert, 1998). These models are proven to be efficient in representing the experimental data. However, they are only verified within specific settings, and exhibit, in some cases, dissimilar patterns. Hence, the exact variables optimized in the brain still remain unclear. Later studies suggest that, instead of a single cost function, the CNS might actually consider a weighted combination of costs during the optimization (Cruse and Brüwer, 1987; Rosenbaum et al., 1995; Desmurget et al., 1998; Wolpert and Kawato, 1998; Gielen, 2009). It has already been verified that the trade-off between the objective (task-related) and the subjective (subject-related) cost functions exists in the CNS (Liu and Todorov, 2007), however, there is still no clear explanation about how the subjective costs are combined in reaching motions. In Berret et al. (2011), this cost combination hypothesis was tested in point-to-bar reaching motions on a vertical 2D plane. An inverse optimal control framework, which was initially proposed in Mombaur et al. (2010) for locomotion planning, was applied to identify the contribution of different cost functions. Though their results support the idea of the combined cost functions, an in-depth analysis on how this combination is formed in 3D reaching motions and whether there is a relationship between the degree of contribution and the reaching task parameters is still missing.

Inverse reinforcement learning (IRL), also sometimes used synonymously with inverse optimal control (IOC), is another line of formulation to find control models, or optimal policies given some demonstrations or observations. However, most of the state-of-the-art methods operate on features rather than raw states, without relying on the dynamical system as a hard constraint on the optimization problem. In essence, the best combination of features, which are extracted during an agent interacting with the environment, is solved for minimizing a pre-defined cost function (Ziebart et al., 2008; Ratliff et al., 2009; Theodorou et al., 2010; Levine et al., 2011; Mainprice et al., 2016). A recent approach by Finn et al. (2016) extends such IRL formulation by tackling the requirement on defining informative features with using neural networks to parameterize the cost function. Essentially, this approach learns non-linear cost functions from user demonstrations, at the same time as learning a policy to perform the task. This formulation can be applied to complex, non-linear cost function representations and high-dimensional problems. However, this is still not directly comparable to solving optimal control problems where the dynamical system is a constraint at each time step, and hence the resulting behaviors are not guaranteed to be generated by the underlying model.

In contrast to creating an optimal control model, another approach to predict human motions is to use data-driven methods. These methods focus more on finding a representation from a given data set (Mainprice and Berenson, 2013; Koppula and Saxena, 2016). Statistical approaches require training data to discover patterns for different arm motions. In that sense, a rigorous and time-consuming data collection process is unavoidable. Other data-driven approaches which do not rely on statistical formulations, e.g., dynamic movement primitives (DMPs) (Ijspeert et al., 2013), require only a minimal set of training data. In an earlier work, we combined optimal control models with DMPs to predict human reaching motion behaviors while avoiding obstacles (Oguz et al., 2016). In that regard, Interaction Primitives (IPs) were developed based on DMPs as a compact representation of a dyadic activity to predict and plan interaction behaviors (Amor et al., 2014). IPs are learned as a distribution over DMP parameters by training on two interacting partners’ trajectories. These IPs encode reciprocal dependencies of dyad movements during the execution of a specific task. The robot then mimics one partner by using the learned model to interact with a human in a similar task. In essence, the learned distributions are conditioned on an observed partial trajectory to predict a human partner’s movement for the rest of the task, which in turn enables the robot to correlate its own motion w.r.t. the human to achieve a successful cooperation. Recently, Environment-adaptive Interaction Primitives (EaIPs) were proposed by extending the IPs with the integration of environmental parameters of the task (Cui et al., 2016). Hence, EaIPs enable inferring movement behavior by conditioning on not only the partner trajectory but also the task and environment related features. However, these are pure data-driven approaches, and thus, they can neither elicit the underlying principles of human interaction movement control, nor provide any means to analyze optimality of observed movements. In addition, our proposed hybrid framework is flexible to integrate such interaction primitives as the data-driven part of the formulation to predict human motions, which can further be integrated into a trajectory optimizer for the robot motion planning in HRI scenarios (Oguz et al., 2017).

Finally, human motor control by the CNS is recognized as a stochastic system (Todorov and Jordan, 2002), thus the variance of the motion should be considered in the trajectory prediction. In Paraschos et al. (2013), a probabilistic movement primitives (ProMPs) approach was proposed with the ability to encode the variance in a general probabilistic framework for representing and learning movement primitives (Schaal et al., 2005). The ProMPs has been successfully implemented in human–robot interaction (Wang et al., 2013) and human–robot collaboration (Maeda et al., 2016a,b) scenarios for controlling the robot motion. For a close cooperation between the robot and human, a precise prediction of the human behavior is essential (Mainprice and Berenson, 2013). However, while predicting human motions with the ProMPs, the integration of the kinematics and dynamics of the human arm is not straightforward. Our work combines an optimal control model with the ProMPs, in order to make use of the advantages from both methods.

## 3 Optimality Criteria for Human Reaching Motions

In this section, we explain the formulation of finding the optimality criteria for human reaching motions in 3D. Many of the influential studies in neuroscience have relied on the hypothesis that the human as a biological entity should minimize a quantitative measure (Engelbrecht, 2001). Based on this, the reaching motion can be formulated as an optimal control problem (OCP), where a given cost function is optimized and used to describe the motion characteristic. Later studies on motor control, learning, and adaptation suggest that instead of a single cost function, a composite of different performance criteria can better explain human behaviors (Berret et al., 2011). In order to identify how these cost functions are combined, an inverse optimal control framework is presented in this section. Through this framework, we attempt to reveal the underlying principles of human reaching behavior while utilizing those models also for predicting human motions.

### 3.1 Model of the Musculoskeletal System

To formulate the reaching motions as an OCP, a representation of the arm dynamics is required and serves as a constraint during the optimization. A widely used approximation of the arm model in 3D is to consider it as articulated rigid bodies. By ignoring the hand movements, a common model of the musculoskeletal system for the arm consists of four degree-of-freedoms (DoFs) (Van der Helm, 1994a,b), where the shoulder joint has three rotations (roll, yaw, and pitch) and the elbow joint has one rotation (pitch). In our experiments, the recorded 3D reaching motions merely use the roll rotation of the shoulder joint, thus it is neglected in our model. This simplification can highly increase the computational efficiency of the OCP, while still preserving enough accuracy on the results. From the classical Lagrangian formalism (Murray et al., 1994), the dynamics of the 3-DoF arm model can be expressed as

(1) $$\tau =M\left(q\right)\ddot{q}+C\left(q,\dot{q}\right)\dot{q}+G\left(q\right),$$

where the variable $q={\left(q_{1},q_{2},q_{3}\right)}^{⊤}$ denotes the joint angles and $\tau ={\left(\tau_{1},\tau_{2},\tau_{3}\right)}^{⊤}$ represents the torques. Time derivatives of ***q***, i.e., $\dot{q}$ and $\ddot{q}$, are the joint angle velocities and joint angle accelerations, respectively. *M*, *C*, and *G* are the inertia matrix, the Coriolis/centripetal terms, and the gravitational vector, respectively. The viscous frictions and elastic properties of the tissues are neglected as they are difficult to estimate. A visualization of the arm model is presented in Figures 2B,C. The upper arm length and the forearm length, as well as the mass, inertia, and distance to the center of mass are defined as described in Lemay and Crago (1996) and Valero-Cuevas et al. (2009). When the arm is in fully stretched out position, *q*_1_, *q*_2_, and *q*_3_ all have zero rotations.

### 3.2 Inverse Optimal Control as a Bi-Level Optimization Problem

The purpose of IOC is to identify the formulation of the OCP, specifically the cost function it optimizes, which best reproduce the observations. A numerical method for solving an IOC problem is to reformulate it as a bi-level optimization problem (Berret et al., 2011). This method relies on the assumption that the optimal cost function is a composite of several plausible basic cost functions. The contribution of each basic cost function is defined through a weight vector, and this weight vector is identified by using the bi-level optimization framework presented in equation (2)

(2) $$\begin{array}{c} \text{Upper}\;\text{level}\;\text{program}:\;\underset{\alpha }{\text{min}}\;\Phi \left(x_{\alpha }^{*},x^{\mathrm{obs}}\right),\\ \;\text{with}\;\sum_{i=1}^{N}\;\alpha_{i}=1,\\ \;⇕\\ \text{Lower}\;\text{level}\;\text{program}:\;\underset{x,u}{\text{min}}\;J\left(x,u|\alpha \right):=\sum_{i=1}^{N}\;\alpha_{i}J_{i},\\ \;\text{s.t.}\;g\left(x,u\right)\leq 0,\;h\left(x,u\right)=0. \end{array}$$

#### 3.2.1 Lower Level Program

The lower level program of the bi-level optimization is a direct OCP (Bertsekas et al., 1995) given by

(3) $$\underset{x,u}{\min}\;J\left(x,u|\alpha \right):=\sum_{i=1}^{N}\alpha_{i}J_{i},\;\text{s.t.}\;g\left(x,u\right)\leq 0,\;h\left(x,u\right)=0.$$

The goal of OCP is to find the optimal trajectory which minimizes a given cost function *J*. Here, *J* is assumed to be a linear combination of *N* basic cost functions *J_i_* (*i* = 1…*N*) which are weighted by the weight vector ***α*** = (α_1_,*α*_2_, …, α_*N*_). The variables *x* and *u* are the vector of system states and control signals, respectively. With above explained arm model, the system states in this work are given as $x^{⊤}=\left(q^{⊤},{\dot{q}}^{⊤},{\ddot{q}}^{⊤}\right)$. Since the joint torques ***τ*** are smoothly generated by muscle contractions (Uno et al., 1989), the control signals are defined as the time derivative of torques $u=\dot{\tau }$. Thus the OCP of reaching motions can be stated mathematically as: *find the admissible system state trajectory* $x_{\alpha }^{*}\left(t\right)$*and control signal trajectory* $u_{\alpha }^{*}\left(t\right)$*in time T, which minimize the cost function J with respect to a given weight vector* ***α****, while satisfying the system dynamics and the task constraints*. For reaching motions, the task constraints contain two parts: the initial condition ***x***(0) = ***x***_s_ and the final condition ***x***(*T*) = ***x**_e_* as the boundary constraints; limitations on joint angles ***q***_min_ ≤ ***q***(*t*) ≤ ***q***_max_ as the inequality constraint. The constraints of joint angle velocities and joint angle accelerations are set to a large range since during the preliminary analysis they are identified to be merely active.

One classical method to solve OCP is to first transform it into a non-linear programming (NLP) problem with constraints, then solve it by using structure exploiting numerical NLP solution methods. In our work, we utilize the multiple shooting method (Diehl, 2011) with ACADO toolkit (Houska et al., 2011) to resolve OCPs.

#### 3.2.2 Selection of Basic Cost Functions

**Figure 3:**
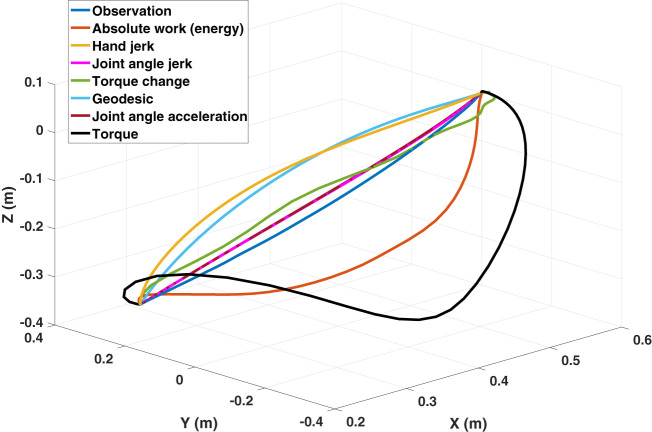
An example of the optimal end-effector trajectories solved from OCPs with respect to different basic cost functions. The variance in their predictions is clear. Only exception is the similarity of the predicted trajectories by minimum joint angle acceleration and the minimum joint angle jerk as they overlap in the figure.

The core part of the IOC framework is to select a set of reasonable basic cost functions. For arm movements, several cost functions were proposed in the past. These cost functions can be categorized into subjective and objective cost functions. Subjective cost functions refer to the decision from a subject, such as the minimum hand jerk (Flash and Hogan, 1985), while objective cost functions are task related. Since the integration of objective cost functions into OCP is difficult, only subjective cost functions are considered in this work. In literature, various subjective cost functions are proven to be useful in explaining human reaching motions (see Table 1). Generally, these cost functions can be grouped as two classes: (a) *kinematic cost functions*: the minimum hand jerk (Flash and Hogan, 1985), the minimum joint angle acceleration (Ben-Itzhak and Karniel, 2008), and the minimum joint angle jerk (Wada et al., 2001) are typical ones and (b) *dynamic cost functions*: the minimum torque change (Uno et al., 1989), the minimum torque (Nelson, 1983), and the minimum absolute work (Nishii and Murakami, 2002; Berret et al., 2008) (also referred as minimum energy throughout this work) belong to this class; and finally the minimum *geodesic* criterion (Biess et al., 2007) is a junction of kinematic and dynamic cost functions, which yields the shortest path in configuration space while taking the kinetic energy into consideration. An example of the optimal end-effector trajectories solved from OCPs with respect to different basic cost functions is given in Figure 3. Among these proposed cost functions, we select five of them as the basic cost functions for IOC, which are the minimum hand jerk *J_HJ_*, the minimum joint angle jerk *J_JJ_*, the minimum torque change *J_TC_*, the minimum energy *J_Enr_*, and the minimum geodesic *J_Geo_*. The minimum joint angle acceleration is ignored since it gives quite similar solution to the minimum joint angle jerk, then the identification between these two cost functions is difficult. In addition, the minimum torque criterion is also neglected because in our preliminary tests we found it has the largest error in describing the reaching motions. Thus, the combined cost function *J* for the direct OCP is defined as

(4) $$J=\alpha_{1}J_{\mathrm{HJ}}+\alpha_{2}J_{\mathrm{JJ}}+\alpha_{3}J_{\mathrm{TC}}+\alpha_{4}J_{\mathrm{Geo}}+\alpha_{5}J_{\mathrm{Enr}}.$$

**Table 1:** Cost functions proposed in literature.

Criterion	Equation
Hand jerk	$J_{\mathrm{HJ}}=\int_{\text{0}}^{T}\;{\overset{\ldots }{x}}^{\text{2}}+{\overset{\ldots }{y}}^{\text{2}}+{\overset{\ldots }{z}}^{\text{2}}\mathrm{dt}$
Joint angle acceleration	$J_{\mathrm{JA}}=\int_{\text{0}}^{T}\;{\dot{q}}_{1}^{2}+{\dot{q}}_{\text{2}}^{\text{2}}+{\dot{q}}_{\text{3}}^{\text{2}}\mathrm{dt}$
Joint angle jerk	$J_{\mathrm{JJ}}=\int_{\text{0}}^{T}\;{\overset{\ldots }{q}}_{\text{1}}^{\text{2}}+{\overset{\ldots }{q}}_{\text{2}}^{\text{2}}+{\overset{\ldots }{q}}_{\text{3}}^{\text{2}}\mathrm{dt}$
Torque change	$J_{\mathrm{TC}}=\int_{\text{0}}^{T}\;{\dot{\tau }}_{\text{1}}^{\text{2}}+{\dot{\tau }}_{\text{2}}^{\text{2}}+{\dot{\tau }}_{\text{3}}^{\text{2}}\mathrm{dt}$
Torque	$J_{\mathrm{Tor}}=\int_{\text{0}}^{T}\;\tau_{\text{1}}^{\text{2}}+\tau_{\text{2}}^{\text{2}}+\tau_{\text{3}}^{\text{2}}\mathrm{dt}$
Absolute work (energy)	$J_{\mathrm{Enr}}=\int_{\text{0}}^{T}\;\left(\sum_{i=\text{1}}^{\text{3}}\;|{\dot{q}}_{i}\tau_{i}|\right)\mathrm{dt}$
Geodesic	$J_{\mathrm{Geo}}=\int_{\text{0}}^{T}\;{\left({\dot{q}}^{⊤}M\dot{q}\right)}^{\text{1/2}}\mathrm{dt}$

*Variables *x*, *y*, *z* are the hand positions in Cartesian space. M denotes the inertia matrix. Corresponding references for the proposed criteria are given as: minimum hand jerk (Flash and Hogan, 1985), minimum joint angle acceleration (Ben-Itzhak and Karniel, 2008), minimum joint angle jerk (Wada et al., 2001), minimum torque change (Uno et al., 1989), minimum torque (Nelson, 1983), minimum absolute work (Nishii and Murakami, 2002; Berret et al., 2008), and minimum geodesic (Biess et al., 2007)*.

One more important issue in combining the basic cost functions, due to the different units, is that the range of the objective values of different cost functions are usually considerably different, thus they cannot directly be equally compared in equation (4). To overcome this problem, we introduce another scalar factor vector ***S***, with the purpose to balance the objective values of selected basic cost functions to the same range. Thus, equation (4) is transformed into

(5) $$J=\sum_{i}\;S_{i}\alpha_{i}J_{i},\;i\in \left\{\mathrm{HJ},\mathrm{JJ},\mathrm{TC},\mathrm{Geo},\mathrm{Enr}\right\}.$$

To obtain the scalar factor vector for a given reaching task, five optimal trajectories $x_{i}^{*}$ with respect to each basic cost function are first computed by solving the corresponding OCPs. Based on the results, the range of the objective value of each basic cost function can be defined through the minimal and maximal values as *Range_i_* = [*J_i,min_*, *J_i,max_*]. Since all selected basic cost functions are integral cost terms and always produce positive values during the optimization, the minimal values are zeros for all cost functions *J_i_*_,min_ = 0. Then the scalar factor vector can be generated directly by comparing the maximal values *J_i_*_,max_. In our experimental data, we found that the minimum joint angle jerk *J_JJ_* tends to have the largest maximal objective value, therefore, we set the scalar factor of the minimum joint angle jerk to 1, then the ratios between other basic cost functions and the minimum joint angle jerk are chosen to be the corresponding scalar factors

(6) $$S_{i}=\frac{J_{i,\text{max}}}{J_{\mathrm{JJ},\text{max}}}.$$

Note that the scalar factor vector varies when at least either the initial condition ***x****_s_* or the final condition ***x****_e_* changes. Thus before running the IOC for each given observation, the scalar factor vector needs to be determined in order to ensure the accuracy of the result.

#### 3.2.3 Upper Level Program

The purpose of the upper level program is to find the optimal weight vector α^*^ which minimizes the distance error between the optimal trajectory $x_{\alpha }^{*}$ obtained from the lower level program and the observation ***x****^obs^*. This optimization problem can be represented as

(7) $$\underset{\alpha }{\text{min}}\;\Phi \left(x_{\alpha }^{*},x^{\mathrm{obs}}\right),\;\;\text{with}\;\sum_{i=1}^{N}\;\alpha_{i}=1,$$

where Φ is a metric which measures the distance error.

Selecting a good metric Φ is crucial in the bi-level optimization framework since it highly affects the decision on the optimal weight vector. The recorded observations are usually the position trajectories in Cartesian space represented by *x*, *y*, *z* coordinates. These observations cannot be directly compared by Φ because, on the one hand, the system states ***x*** are defined as joint angles, on the other hand, the position trajectories usually contain uncertainties, which come from: (1) the error from the torso movement and (2) the difference between the subject’s actual arm length and the defined musculoskeletal system’s arm length. No consistent results can be derived if a direct comparison to the position trajectories is implemented in Φ.

To address this problem, we transform the recorded position trajectories to the *relative position trajectories in arm model coordinate system* through the following steps:

1. Record the Cartesian position trajectories of the shoulder joint ***t****_s_* = (*t_s,x_*,*t_s,y_*,*t_s,z_*), the elbow joint ***t****_e_* = (*t_e,x_*,*t_e,y_*,*t_e,z_*), and the wrist joint ***t****_w_* = (*t_w,x_*, *t_w,y_*, *t_w,z_*).
2. Derive the observed joint angle trajectory through the arm geometry as ***q***^obs^ = *G*(***t****_s_*, ***t****_e_*, ***t****_w_*). Since the roll rotation of the shoulder joint is neglected in our work, the translation function *G* can be easily obtained.
3. Compute the relative position trajectory (end-effector trajectory) in arm model coordinate system by using the kinematic relationship of the proposed arm model as ***t****^obs^* = δ(***q****^obs^*), where δ represents the function of the forward kinematics.

The relative end-effector trajectory ***t****^obs^* eliminates the error caused by different arm lengths and the torso movements, thus it can be compared to the solution calculated from the lower level program.

Based on the feature compared in Φ, two different types of the distance metric can be formulated: one is *the joint angle metric*, where the observed joint angle trajectory ***q****^obs^* is compared to the optimal system states trajectory $x_{\alpha }^{*}$, which also contains the joint angle trajectory $q_{\alpha }^{*}$; another is *the end-effector trajectory metric*, where at first the optimal end-effector trajectory $t_{\alpha }^{*}$ is computed from the optimal joint angle trajectory $q_{\alpha }^{*}$ by using the same forward kinematics function δ, then the distance error is calculated between the relative end-effector observation ***t****^obs^* and $t_{\alpha }^{*}$.

In our preliminary tests, we found that the end-effector trajectory metric has a better performance than the joint angle metric. Possible reason is that the three joint angles actually have different degrees of influence on the reaching motions (Nguyen and Dingwell, 2012). However, it is not straightforward to determine the contribution of different joint angles, which could introduce further uncertainties and errors. Similar problem also occurs when combining the joint angle metric and the end-effector metric, since they have different units and it is difficult to balance them into the same range. Therefore, in our work, the distance metric of the upper level program only considers the end-effector trajectories, which can be treated as comparing two 3-dimensional signals. The dynamic time warping (DTW) algorithm (Vintsyuk, 1968) is implemented to calculate the distance error. In time series analysis, DTW is used for measuring the similarity between two temporal sequences which may vary in speed. The sequences are first warped in the time dimension and then compared to each other. With this, equation (7) can be stated as

(8) $$\underset{\alpha }{\text{min}}\;\Phi \left(x_{a}^{*},x^{\mathrm{obs}}\right):=\underset{\alpha }{\text{min}}\;D\left(t_{\alpha }^{*},t^{\mathrm{obs}}\right),$$

where *D* denotes the DTW calculation.

To solve equation (8), common gradient-based methods and stochastic optimization algorithms are not applicable because of two reasons. First, the metric Φ is non-differentiable with respect to the weight vector α; second, before each calculation of Φ, a direct OCP must be solved in advance, thus it usually takes a few minutes for one evaluation. Specifically, the stochastic optimization algorithms (e.g., particle swarm optimization (Eberhart and Kennedy, 1995)) are also not suitable here, since they require more samples which will result in infeasible computation time. Hence, the upper level program is optimized by a robust derivative-free optimization (DFO) method. Here, we use the method called CONDOR (Berghen and Bersini, 2005) for COnstrained, Non-linear, Direct, parallel optimization, which is a parallel extension of the Powell’s method (Powell, 2004) based on the trust region algorithm (Sorensen, 1982). Through a local approximation of Φ, it can find the optimal solution more efficiently than the common pattern search and stochastic optimization techniques. To reduce the computation time, the initial value of ***α*** should be set properly before the optimization. Since among the five elements of α only four are actually independent, and OCPs corresponding to the costs *J*(***α***) and *J*(λ***α***), λ > 0 are identical, a practical strategy is to fix one element to one and then adjust the remaining components with respect to it (Mombaur et al., 2010). As all the basic cost functions are scaled into the same range, the value of other components can be restricted to stay in [0,1]. During the optimization process, if any element is found larger than one, the optimization should be restarted with setting this element to one. In our experimental data, setting the weight of joint angle jerk to one gives the best results in most cases. After around 100 iterations, the algorithm converges to a local minimum. Note that due to the high non-linearity of the problem formulation, the global minimum is not available in the bi-level optimization (Albrecht et al., 2012). In order to get more accurate results while keeping a reasonable computation time, we set the initial value of α to (0.5, 1, 0.5, 0.5, 0.5) and solve it three times with different initial search radii (Powell, 2004) as 0.15, 0.3, and 0.45, so that most range is covered within three IOC calculations. The one results in the minimum distance error is considered as the final optimal weight vector α* and is normalized for later analysis.

### 3.3 Representation of the Reaching Motions

From the IOC formulation, we acquire a weighted combination of cost functions, which specifies the contribution of each model for the realization of a reaching motion. For each specific motion behavior, one composite model needs to be found. However, we can only have a limited number of different composite models, due to the computational time limit. To utilize the composite model in general cases, a mapping from the motion parameters to the contribution of cost functions is required. According to the results of the initial experiment we conducted, which is detailed out in Section 5.1.3, a correlation between the initial and the final joint angles (***q****_s_*,***q****_e_*) and the optimal weight vector α* is identified. Here, we use the Gaussian Process Regression (GPR) model (Rasmussen and Williams, 2005) to represent the mapping as

(9) $$\alpha^{*}=\mathrm{GPR}\left(q_{s},q_{e}\right),$$

where *GPR* denotes the GPR model. The optimal weight vector returned by the GPR model is a distribution with mean and variance. Note that the GPR model can be replaced by other similar stochastic models, but we find that the GPR model is more suitable in our case since it requires less data. This GPR model provides a connection between the IOC formulation and the ProMPs in our hybrid online prediction framework.

## 4 Hybrid Online Prediction Framework

In literature, many prediction methods for human motion were proposed. Among them, two classes of the methods are widely used: (1) *model-based methods*, where a motion model is created based on minimizing a criterion, such as the minimum hand jerk model (Flash and Hogan, 1985), the minimum joint angle jerk model (Wada et al., 2001), and the minimum variance model (Harris and Wolpert, 1998). Then the solution to the model is considered as the prediction; (2) *data-driven methods*, where a set of data (observations) should be available before building a generative model for predicting human motions. The characteristic of the motion can be learned from the data and then the prediction is generated by reproducing this characteristic and in some cases with variation. Gaussian Mixture Models (McLachlan and Basford, 1988; Calinon et al., 2010), dynamic movement primitives (Ijspeert et al., 2013), and probabilistic movement primitives (Paraschos et al., 2013) are typical data-driven methods. In this section, we propose a hybrid online prediction framework for reaching motions by combining a model-based method and a data-driven method. Instead of using the motion model with single cost function, a composite model is obtained by the IOC framework. In order to deal with the motor variability of the reaching motion (Todorov and Jordan, 2002), this composite model is combined with the ProMPs. ProMPs are selected due to both their capability on learning a model with a very small amount of observations (in our experiments 5–10 samples seem to be enough), and also their computational efficiency for rolling-out predictive trajectories online. Especially, it is known that GMMs tend to perform poorly in high-dimensional spaces when few data points are available (Calinon, 2016). In the rest of this section, first a brief explanation of the ProMPs is presented, then a comparison between the predictions of the composite model and the ProMPs is discussed. Finally, the hybrid prediction framework is explained in detail.

### 4.1 Probabilistic Movement Primitives

The ProMPs is a probabilistic formulation for movement primitives. It is able to capture the variance information of trajectories and represent the behavior in stochastic systems. Given a discrete trajectory ***X*** = {*x_t_*}, *t* = 0…*T* defined by states *x_t_* over time *T*, a weight vector ***ω*** is used to represent the trajectory as

(10) $$y_{t}={\left[x_{t},{\dot{x}}_{t}\right]}^{⊤}=\Phi_{t}^{⊤}\omega +\epsilon_{y},$$

where $\Phi_{t}=\left[\phi_{t},{\dot{\phi }}_{t}\right]$ denotes the *n* × 2 dimensional time-dependent basis matrix for states *x_t_* and the velocities ${ẋ}_{t}$. *n* is the number of basis functions and $\epsilon_{y}∼N\left(0,\Sigma_{y}\right)$ is zero-mean independent and identically distributed Gaussian noise. The mean of the trajectory can be obtained by weighting Φ*_t_* with ***ω***. The probability of observing a trajectory ***X*** with a given ***ω*** is represented by a linear basis function model as

(11) $$p\left(X|\omega \right)=\prod_{t}\;N\left(y_{t}|\Phi_{t}^{⊤}\omega ,\Sigma_{y}\right).$$

In order to capture the variance, a Gaussian distribution $p\left(\omega ;\theta \right)=N\;\left(\omega |{\mu_{\omega }}_{,}\Sigma_{\omega }\right)$ over the weight vector ***ω*** is introduced with parameters ***θ*** = {***μ***_***ω***_, **Σ**_***ω***_}. Then the distribution of ***y****_t_* at time *t* is given by

$$\begin{array}{llll} p\left(y_{t};\theta \right) & =\int N\;\left(y_{t}|\Phi_{t}^{⊤}\omega ,\Sigma_{y}\right)\;N\;\left(\omega |\mu_{\omega },\Sigma_{\omega }\right)d\omega \; & & \;\\ & =N\;\left(y_{t}|\Phi_{t}^{⊤}\mu_{\omega },\Phi_{t}^{⊤}\Sigma_{\omega }\Phi_{t}+\Sigma_{y}\right).\; & \text{(12)} \end{array}$$

With equation (12), the mean and the variance of the states for any time point *t* can be derived. If a set of observations is available, the parameters ***θ*** can be learned by using the maximum likelihood estimation (Lazaric and Ghavamzadeh, 2010). In reaching motions, the distribution *p*(***ω***; ***θ***) can be considered as a representation of the motor variability. For more details of the ProMPs please refer to Paraschos et al. (2013).

### 4.2 Comparison Between the Composite Model Prediction and the ProMPs Prediction

Both the composite model formulation and the ProMPs framework have clear advantages and drawbacks, but they are also complementary. By combining them into a hybrid prediction framework, the advantages of both methods can be exploited at the same time (Table 2).

**Table 2:** Different perspectives of the composite model prediction and the ProMPs prediction.

Perspective	Composite model	ProMPs
Underlying principle	Yes	No
Optimality	Yes	No
Computation time	High	Low
Motor variability	No	Yes

The composite model represents the underlying principles of reaching motion control. Several motion models have been proven to be accurate in describing the movements, such as the minimum hand jerk model on some tasks, and the minimum torque change model on others, in 2D reaching motions. The composite model we proposed inherits those capabilities and extends it to the 3D reaching motions. It helps us explain how humans execute and control their reaching motions, while extracting such information from the data-driven methods is not trivial. However, the biggest obstacle in implementing the composite model prediction in online case is the computation time. Before rolling out the optimal trajectory, an OCP needs to be solved, which usually takes several minutes, even when the state-of-the-art solvers are used (Diehl, 2011). However, in real world settings, the reaching motions take no longer than a few seconds, thus the data-driven methods are more suitable in the online case, as they are computationally more efficient.

Another important reason of using the ProMPs as the data-driven method in the hybrid prediction framework is that it allows describing the motor variability given sample demonstrations (Paraschos et al., 2013). As explained in Todorov and Jordan (2002), human motor control is a stochastic system with signal-dependent noise (Harris and Wolpert, 1998), thus reaching motions are expected to show variance. Since it is not straightforward to consider the variance within an IOC problem, we formulate our composite model as a deterministic OCP. On the other hand, as the ProMPs formulation employs a probabilistic function to represent the motion, the obtained model is not a single trajectory but a distribution of trajectories. Hence, while the composite model describes an optimal average behavior as an initial guess, the ProMPs enables capturing the multiplicative noise due to motor control. However, to understand the control model due to such noise, the model-based IOC computation and a follow-up GPR update is still required.

### 4.3 Prediction Framework

**Figure 4:**
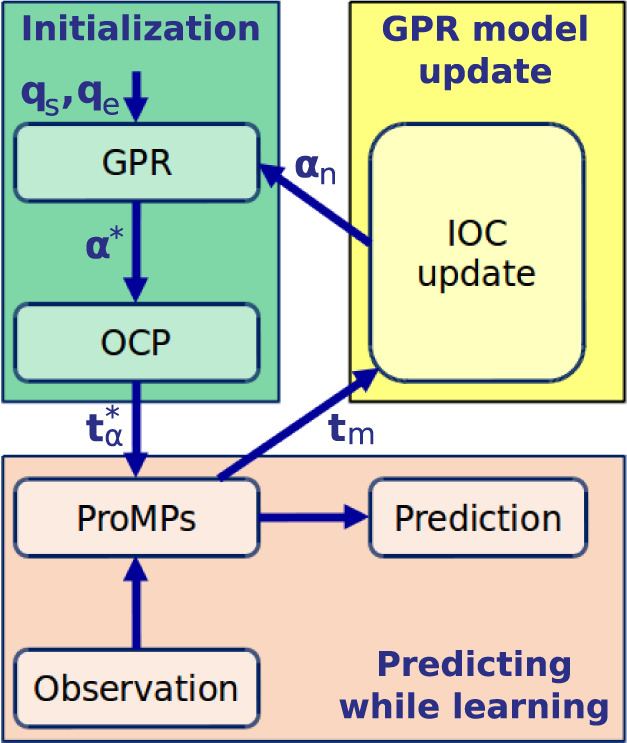
Overview of the prediction framework (upper right in Figure 1). ***q***_s_ and ***q***_e_ are the initial and final joint angle configurations. α* is the estimated optimal weight vector and $t_{\alpha }^{*}$ is the corresponding optimal solution from OCP. ***t****_m_* denotes the mean of the converged trajectory distribution extracted from the ProMPs, α*_n_* is the new obtained optimal weight vector, which is used to update the GPR model.

The idea of the hybrid prediction framework is, for a given reaching task, to use the composite model to generate the initial training data for the ProMPs. Then in the online phase, the ProMPs can rollout predicted trajectories with high efficiency while also learning the variance by using each motion observation as new training data. After several observations, the parameters of the ProMPs converge (the details is explained in Section 5.2.2), then the mean of the converged trajectory distribution is calculated to update the composite model. An overview of the framework is given in Figure 4, and the details of this hybrid model are explained next.

#### 4.3.1 Initialization With the Composite Model

Usually for a given reaching task, the starting position and the target position are known. Through the inverse kinematics, the initial joint angle configuration ***q***_s_ and the final joint angle configuration ***q***_e_ can be approximated. By using the GPR model trained on the IOC results, a distribution of the estimated optimal weight vector is available. However, due to the limited amount of data for training the GPR model, the variance cannot be learned accurately. Thus, only the mean value of the distribution α* is used here. After solving the OCP with respect to α*, the optimal joint angle trajectory $q_{\alpha }^{*}$ and its corresponding end-effector trajectory $t_{\alpha }^{*}$ are obtained. $t_{\alpha }^{*}$ is considered as the training data for the ProMPs. As the OCP gives a deterministic solution, no variance information can be derived. Hence the ProMPs is initialized by learning the parameters from the optimal trajectory $t_{\alpha }^{*}$, while setting the variance to a large value.

#### 4.3.2 Predicting While Learning

During online prediction, a trajectory along with the variance for each time point is generated by the ProMPs. This variance information is useful for human–robot interaction scenarios where the robot should also consider the uncertainties of human behaviors. The observations recorded during the prediction are utilized to update the ProMPs to get a more accurate representation of the variance. After each movement, the observation is added to the data storage which contains all the previous observations. Subsequently, the ProMPs update their parameters from the new data storage. With the incorporation of each motion observation, parameters of the ProMPs as well as the variance information converge.

#### 4.3.3 GPR Model Updating

Once the ProMPs system becomes stable, the mean of the converged trajectory distribution ***t****_m_* can be extracted. This trajectory can be considered as the average behavior of the recorded subject for this reaching task. Then in a separate updating process, ***t****_m_* is used by the IOC framework to get the corresponding optimal weight vector. The new optimal weight vector ***α****_n_* is used to update the GPR model. Therefore, with more information returned from the real recordings, the GPR model also becomes more accurate in describing the mapping from the initial and final joint angles to the optimal weight vector.

### 4.4 Motor Variability and Interpersonal Variance

The motor variability is essential in describing human behaviors (Todorov and Jordan, 2002), as it can be considered as the uncertainties of human motions (e.g., the noise in motor command). It represents the fact that for a given reaching task, even the same subject is expected to execute the motion in slightly different trajectories. This phenomenon has been reported in sensorimotor control by demonstrating such variability on observed experimental data for a multitude of tasks, e.g., locomotion (Winter, 1989), writing (Bernstein, 1967), pointing (Tseng et al., 2002), reaching (Haggard et al., 1995), and grasping (Cole and Abbs, 1986). Usually for simple tasks, this difference is not large and can be modeled as a probabilistic distribution (Knill and Pouget, 2004; Koppula and Saxena, 2013). However, such probabilistic models cannot explain the underlying cause of observing such motor variability, which is known to be due to additive and multiplicative noise in the motor control and is treated as the intra-subject variance in this work. Apart from the motor variability, there are also motion behavior differences between subjects (Vu et al., 2016a), which we call interpersonal variance in this work. The existence of such a disparity can be verified through the contribution of basic cost functions, as shown in the next section. The interpersonal variance suggests that humans plan their motions in a personal way, which reflects the dissimilarity of the control structure due to learning and adaptation effects, along with biomechanical differences. Thus, the updated GPR model from the hybrid prediction framework is actually a person-specific model.

## 5 Experiments and Results

In this section, two experiments and their corresponding results are presented. One is designed for the IOC framework with the purpose of understanding the characteristics of human reaching motions, and the other is used to test the performance of the hybrid online prediction framework.

### 5.1 Experiment for the IOC Framework

To cover the reaching motions in a relatively large range, we designed an experiment for point-to-point reaching tasks consisting of 12 starting postures and 9 target regions. The recorded trajectories were analyzed through the IOC framework. Based on the obtained optimal weight vectors, we find that the contribution of basic cost functions has a relationship with the initial and final joint angle configurations. Besides, the composite cost function is proven to have less error in describing the reaching motions in almost all tasks compared to the single cost models. This result encourages us to use the composite model in the prediction rather than a model with single cost function. In the rest of this subsection, at first the details about the experimental setup are presented, then the results from the IOC framework are discussed.

#### 5.1.1 Experimental Setup and Data Collection

**Figure 5:**
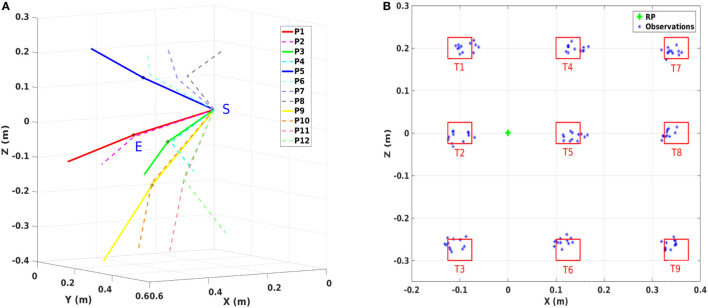
Experimental setup. **(A)** Visualization of 12 starting joint angle configurations. P1 to P4 are the postures with *q*_1_ in the middle (no rotation), while P5 to P8 are the postures with *q*_1_ in the up region and P9 to P12 with *q*_1_ in the down region. **(B)** Target areas on the board surface. RP denotes the reference point. Observations are the actual positions where the 108 averaged trajectories terminate on the board surface.

A visualization of the experimental setup is presented in Figure 2A. Participants were required to sit before a board which was placed vertical to the ground surface. Nine target areas and one reference point were marked on the board as square regions with the side length equal to 5 cm. The distances between the target areas and the reference point are shown in Figure 5B. Before the experiment, the sitting height of the participant was adjusted by setting a straight line between the reference point and the center of the shoulder joint vertical to the board surface. Then the distance between the center of the shoulder joint and the board surface was selected as 80% of the arm length. These distances were chosen to ensure that the participants can reach all nine targets easily without moving their torso.

Since we want to cover a large range of reaching motions, every participant was asked to reach the nine targets from 12 different starting arm postures. According to the joint angle limits we defined in the arm model, these starting postures were chosen from the combination of three different *q*_1_, two different *q*_2_, and two different *q*_3_ (3 × 2 × 2 = 12) configurations (see Table 3). As shown in Figure 5A, the pitch rotation of the shoulder joint *q*_1_ is selected as three configurations: up, middle, and down, respectively. The yaw rotation of the shoulder joint *q*_2_ and the rotation of the elbow joint *q*_3_ are chosen from the stretched to the side configurations and a configuration in the middle of the joint angle limits. With nine targets for each starting posture, 108 (12 starting postures × 9 targets) cases of the reaching motions were considered in the experiment.

**Table 3:** Actual starting joint angle configurations.

Posture	q_1,S_(^∘^)		q_2,S_ (^∘^)		q_3,S_ (^∘^)	
	Mean	SD	Mean	SD	Mean	SD
P1	10.95	5.01	6.58	4.66	12.72	3.49
P2	11.21	5.73	8.78	9.47	33.39	6.51
P3	11.93	3.70	31.90	5.82	13.15	3.74
P4	13.00	6.37	34.45	6.80	37.92	8.53
P5	−22.29	5.21	12.46	5.18	14.11	3.86
P6	−23.47	5.71	15.82	6.41	37.88	7.51
P7	−22.89	5.33	37.31	7.91	16.10	5.09
P8	−23.64	5.66	41.07	8.42	35.75	7.88
P9	42.15	6.16	6.98	7.43	12.28	4.58
P10	40.22	4.40	7.08	5.28	35.40	5.59
P11	35.36	5.09	36.14	5.61	10.06	5.76
P12	35.14	5.45	36.88	6.85	43.44	6.69

*P1 to P12 are the 12 predefined starting postures. *q*_1,S_, *q*_2,S_, and *q*_3,S_ are the three starting joint angles with respect to the stretched out posture. The values are computed by using all 15 subjects’ data*.

Before the recording, the arm posture was determined by measuring all three joint angles to ensure all participants shared the same starting joint angle configuration. The participants were given the following instructions. First, in order to discard the decision-making process of target selection, the subject needs to reach the nine targets in a fixed order as from target one to target nine. Second, the participant should strictly put his arm in the previously set starting posture before executing the follow-up reaching task. A set of special reference tools were prepared and put beside the participants. These tools consist of two bars and their end points indicate the positions of the elbow and wrist joints for the given starting posture. Reference tools were placed in appropriate positions so that during the reaching motion they do not block any potential motion trajectory. Third, in order to eliminate the effect of locating targets during the movement, before the execution of the reaching tasks, the participants should look at the targets rather than the reference tool. Fourth, the participants were told to avoid using the roll rotation of the shoulder joint, which is ignored in our arm model. In addition, all participants were trained before the experiments to get familiar with the setup and the task. If any unintended motion was detected during the recording, corresponding tasks were executed again. Between each starting posture, enough rest time was provided for avoiding fatigue. To reduce the noise, every target in every starting posture was reached two times, thus a total of 216 (108 cases × 2 times) trajectories were recorded for one participant.

The data were collected from fifteen subjects (11 males, age: 27 ± 4; weight: 67 ± 9 kg, height: 172 ± 5 cm) who all gave written informed consent for their participation. All the participants were right-handed with normal vision ability. None of them received any information about the purpose of the experiment. The study was approved by the ethics committee of the Technical University of Munich School of Medicine. The reaching motions were recorded by the multicamera motion capture system Qualisys at a frequency of 250 Hz. With the built-in filter function, the smooth position trajectories of the shoulder, elbow, and wrist joints can be directly obtained from the tracking system and used for the IOC calculations.

#### 5.1.2 Average Motion Behavior

In our IOC framework, we are interested in the control structures for the human reaching motion behavior in a general sense, rather than the individual differences. We also intend to provide a base model to be extended for person-specific motion behaviors during prediction. Hence, we compute the average trajectories from all 15 subjects, and the IOC problems are solved for these trajectories. Besides, the averaging process also saves a lot of computation time. Since the IOC calculation for one trajectory roughly takes 4 h, the analysis on all 1,620 (15 subjects × 108 cases) trajectories would require an immense amount of time. Table 3 gives the mean values and the SDs of 12 starting joint angles calculated from all subjects’ data. The SDs indicate that for the same starting posture, all subjects started their reaching motions with a relatively small joint angle difference, which enables the feasibility of averaging the trajectories. If not mentioned explicitly, all the IOC results presented in the following part are based on the averaged trajectories.

#### 5.1.3 Results for the IOC Framework

After the IOC calculations, we obtained one optimal weight vector for each reaching task. The contribution of basic cost functions in 108 different cases are analyzed next.

##### 5.1.3.1 Performance in Describing the Reaching Motions

**Figure 6:**
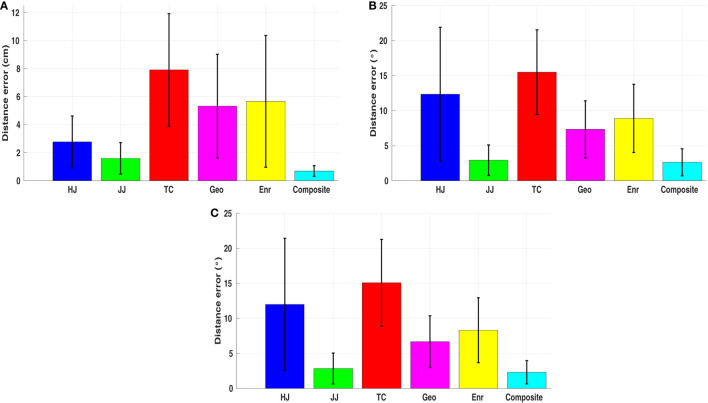
Average distance error over all reaching tasks. HJ, JJ, TC, Geo, Enr, and Composite are the hand jerk, the joint angle jerk, the torque change, the geodesic, the energy, and the composite cost function, respectively. Mean values and the SDs of the errors for each cost function are presented. **(A)** Distance error measured by comparing the end-effector trajectories. **(B,C)** Distance error measured by comparing the joint angle trajectories with and without considering target one.

To verify the performance of the composite model, the optimal trajectory solved with it is compared to the optimal trajectories computed for each single basic cost function. The distance error between each optimal trajectory and the average motion behavior is measured through the DTW-based comparison separately. The results show that, almost for all cases, the composite model has a better performance in describing the reaching motions. Even though the distance metric we used in the upper level program of the IOC framework only considers the end-effector trajectory, the composite model still has less errors in the joint angle trajectories. Figure 6 presents the distance error averaged from all 108 cases. The p-test results indicate that, there are significant decreases on the distance error when comparing the composite model to all other five basic cost functions (p*_i_* < 0.0001, *i* = 1, … , 5). In joint angle trajectories, except the minimum joint angle jerk cost function (p = 0.1813), we still observe significant decreases (p < 0.0001). The reason is, in 3D reaching motions, the observed joint angle trajectories are bell-shaped, which are quite close to the results derived from the minimum joint angle jerk cost function, especially when the reaching motion enforces approaching the joint angle limits (e.g., reaching target one). After we removed the cases of reaching target one in the comparison, there is still a significant decrease (p < 0.05), now for all the cases, on the distance error in describing the joint angle trajectories with the composite model. Furthermore, it should be noted that, optimizing only dynamics related cost functions leads to inconsistent arm trajectories in terms of joint and Cartesian displacements (a single case is shown in Figure 3). By contrast, even though maximizing smoothness in joint space (angel jerk, i.e., kinematic cost) was efficient to fit the angular and Cartesian displacements, it is reported by Vu et al. (2016b) that it fails to describe the movement in torque space accurately. It appears that the composite optimality criterion comprising different biomechanical properties is the only model that can explain both kinematic and dynamic aspects of the reaching behaviors.

##### 5.1.3.2 Influence of the Initial and Final Conditions

In order to get a deeper understanding of the human reaching motions, an analysis on identifying the possible factors which influence the contribution of basic cost functions is performed. We conduct the *N*-way independent analysis of variance (ANOVA) on our results with four factors, the three starting joint angles *q*_1,*s*_,*q*_2,*s*_,*q*_3,*s*_ and the target index *T*. As ANOVA checks the importance of one or more factors by comparing the response variable means at different factor levels, the results obtained can be utilized to identify the factors which have statistical significant influence on the examined variable. In Table 4, we list the corresponding results from our ANOVA analysis when selecting the response variable as the contribution of five different basic cost functions as well as the sum of dynamics related cost functions (the minimum torque change + the minimum energy), respectively.

**Table 4:** Results of ANOVA tests.

RV:factor	Sum.Sq.	Mean.Sq.	F	p
HJ:*q_1,S_*	0.9624	0.4812	19.5487	0.0000
HJ:*q_2,S_*	0.1872	0.1872	7.6063	0.0078
HJ:*q_3,S_*	0.0068	0.0068	0.2750	0.6020
HJ:*T*	0.1635	0.0204	0.8303	0.5796
JJ:*q_1,S_*	0.4115	0.2058	10.7701	0.0001
JJ:*q_2,S_*	0.0150	0.0150	0.7830	0.3799
JJ:*q_3,S_*	0.0176	0.0176	0.9223	0.3409
JJ:*T*	0.2026	0.0253	1.3255	0.2494
TC:*q_1,S_*	0.1005	0.0503	12.7500	0.0000
TC:*q_2,S_*	0.0081	0.0081	2.0525	0.1573
TC:*q_3,S_*	0.0004	0.0004	0.1092	0.7423
TC:*T*	0.0603	0.0075	1.9122	0.0753
Geo:*q_1,S_*	0.1202	0.0601	3.0653	0.0543
Geo:*q_2,S_*	0.0056	0.0056	0.2844	0.5959
Geo:*q_3,S_*	0.0232	0.0232	1.1812	0.2816
Geo:*T*	0.0894	0.0112	0.5702	0.7980
Enr:*q_1,S_*	0.2760	0.1380	7.7557	0.0010
Enr:*q_2,S_*	0.1525	0.1525	8.5667	0.0049
Enr:*q_3,S_*	0.0331	0.0331	1.8596	0.1779
Enr:*T*	0.2721	0.0340	1.9113	0.0755
Dyn:*q_1,S_*	0.6702	0.3351	19.3833	0.0000
Dyn:*q_2,S_*	0.2308	0.2308	13.3516	0.0006
Dyn:*q_3,S_*	0.0411	0.0411	2.3760	0.1287
Dyn:*T*	0.3356	0.0420	2.4267	0.0246

*RV denotes the response variable, selected as the contribution of each basic cost function (HJ: hand jerk, JJ: joint angle jerk, TC: torque change, Geo: geodesic, Enr: energy) and the dynamic related cost functions (Dyn: dynamics, which is the sum of the minimum torque change and the minimum energy). Four variables are considered as the factors, which are the three starting joint angles *q_1,S_*, *q_2,S_*, *q_3,S_* and the target index *T*. RV:factor indicates the ANOVA result of the influence of the factor on the corresponding response variable (e.g., HJ:*q_1,S_* means the influence of *q_1,S_* on the contribution of the minimum hand jerk cost function). Sum.Sq. and Mean.Sq. are the sum of squares due to each source and the mean squares for each source, respectively. F is the *F*-statistic, which is the ratio of the mean squares. p is the *p*-values, which represents the probability that the F-statistic can take a value larger than a computed test-statistic value. Other ANOVA results (e.g., the degree of freedom) are not listed here*.

From ANOVA analysis, it can be concluded that the starting joint angles of the two shoulder rotations have influences on the contributions of the cost functions: *q*_1,*s*_ has influence on the contribution of the hand jerk (F(2,58) = 19.5487, p < 0.0001), the joint angle jerk (F(2,58) = 10.7701, p < 0.001), the torque change (F(2,58) = 12.7500, p < 0.0001), the energy (F(2,58) = 7.7557, p < 0.001), and the dynamics (F(2,58) = 19.3833, p < 0.0001); while *q*_2,*s*_ has influence on the hand jerk (F(1,58) = 7.6063, p < 0.01), the energy (F(1,58) = 8.5667, p < 0.01), and the dynamics (F(1,58) = 13.3516, p < 0.001). For the target position, only the dynamics is affected (F(8,58) = 2.4267, p < 0.05). Finally, the starting joint angle of the elbow rotation *q*_3,*s*_ has no influence on the contribution of basic cost functions (all p > 0.05).

**Figure 7:**
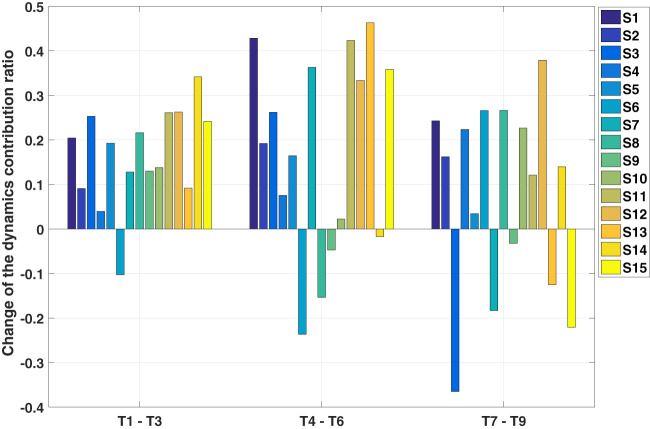
Results of the individual analysis. S1 to S15 represent 15 subjects. The change of the dynamics related cost is derived by subtracting *C_top_* from *C_bot_*, where *C_top_*, *C_bot_* are the contribution of dynamics related cost for targets in the top and bottom row, respectively. (e.g., T1–T3 means subtracting the contribution of the dynamics to the target three from to the target one).

In order to identify how the target position, which can be expressed by the three final joint angles *q*_1,*E*_,*q*_2,*E*_,*q*_3,*E*_, affects the contribution of the dynamics, an individual analysis is conducted on the trajectories of each subject with one starting posture (fully stretched out posture P1) and six targets (top row: T1, T4, and T6, bottom row: T3, T6, and T9). Thus 90 (15 subjects × 6 trajectories) IOC calculations are performed. Then p-test is utilized to find if there is a significant difference between different final joint angles. The results suggest that only *q*_1,*E*_ has influence on the contribution of the dynamics related cost, which indicates that only the height of the targets matters. This can be verified in Figure 7, where we compare the contributions of the dynamics related cost between two sets of targets (top vs bottom row). From these results, the interpersonal variance can also be observed, where the changes are different for each subject, and sometimes this difference can be considerably large.

##### 5.1.3.3 Transition Between Different Reaching Tasks

**Figure 8:**
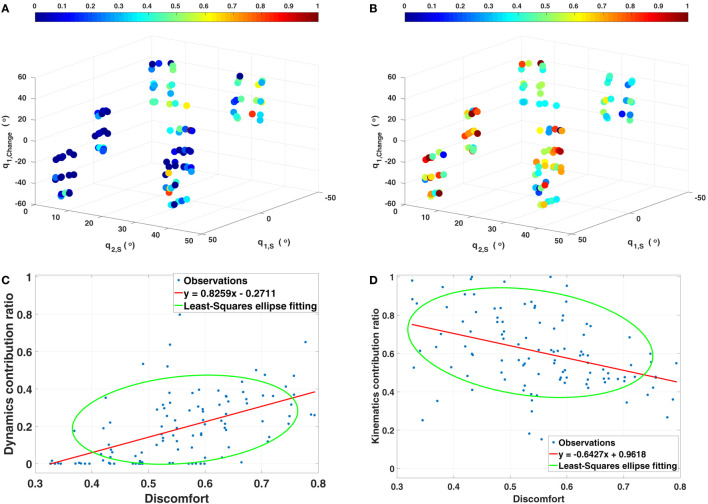
Transition between different cases. **(A,B)** Contribution of the dynamics (left) and the kinematics (right) related costs with respect to three factors. *q*_1_*_,S_*, *q*_2_*_,S_* are the two starting joint angles of the shoulder rotations, *q*_1_*_,Change_* is the change between the final and the initial angle of the pitch rotation of the shoulder joint. The colors indicate the contribution ratio of corresponding cost. **(C,D)** Relationship between the proposed discomfort metric and the contribution of the dynamics and the kinematics related costs. Red lines are the linear regression models created based on the discomfort value with respect to the optimal weights $\left(\beta_{1}^{*},\beta_{2}^{*}\right)$. Another least-squares ellipse fitting is also presented to demonstrate the trend with variance.

According to the previous results, three factors are identified to be related to the contribution of basic cost functions, which are the two starting joint angles of the shoulder joint *q*_1,*S*_,*q*_2,*S*_ and the change of the pitch rotation of the shoulder joint *q*_1,_*_Change_ *= *q*_1,*E*_ −* q*_1,*S*_. In order to identify how exactly these factors affect the contribution, two 3D scatter plots are given in Figures 8A,B. Considering the musculoskeletal loading as the criterion to describe the comfortableness of the reaching motions (Kee and Lee, 2012; Zenk et al., 2012), the fully stretched down posture can be treated as the most comfortable posture. Then the more rotations required to execute the reaching tasks from the fully stretched down posture, the more uncomfortable the motion is. It can be observed that, for comfortable reaching motions (left-down region of the figures), the dynamics related cost function has less contribution while the kinematics has higher, compared to the uncomfortable reaching tasks (right-up region of the figures), where the opposite trend is observed. Based on this, we propose a *discomfort metric* by combining the three factors along with their corresponding joint angle limits as

(13) $$\mathrm{Dis}=\left(\frac{90-q_{1,S}}{180}\right)+\beta_{1}\;\frac{q_{2,S}}{180}+\beta_{2}\;\left(\frac{q_{1,\mathrm{Change}}}{180}\right),$$

where *Dis* denotes the discomfort value calculated by a linear combination of the three factors by using the weights β_1_ and β_2_. Then for a given pair of weights (β_1_, β_2_), a set of discomfort values can be derived for all 108 reaching tasks *Dis_i_* (*i* = 1…108). Each discomfort value has its corresponding contribution value of the dynamics related cost function *C_i_* (*i* = 1…108), hence a simple linear least square regression model can be created from the data set (*Dis_i_*, *C_i_*) (*i* = 1…108) as

(14) $$y=\theta_{1}+\theta_{2}x.$$

By changing the weights, different linear regression models *y_β_*_1,_*_β_*_2_ are obtained. The coefficient of determination (Ross, 2014) *R*^2^ for each model is given by

(15) $$R_{\beta_{1},\beta_{2}}^{2}=1-\frac{\sum_{i=1}^{108}\;{\left(C_{i}-y_{i,\beta_{1},\beta_{2}}\right)}^{2}}{\sum_{i=1}^{108}\;{\left(C_{i}-\bar{C}\right)}^{2}},$$

where *C_i_* is the actual contribution value, *y_iβ_*_1,_*_β_*_2_ represents the calculated contribution value from the linear regression model *y_β_*_1,_*_β_*_2_, $\bar{C}$ is the mean value of *C*. *R*^2^ measures of how well a model can represent the data, and falls between 0 and 1. The higher the value of *R*^2^, the better the model is at predicting the data. Therefore, the optimal pair of the weights is derived by maximizing *R*^2^

(16) $$\left(\beta_{1}^{*},\beta_{2}^{*}\right)=\underset{\beta_{1},\beta_{2}}{\max}\;R_{\beta_{1},\beta_{2}}^{2}.$$

Solving equation (16) with respect to the contribution of the dynamics yields the optimal weights as $\beta_{1}^{*}=0.8150$ and $\beta_{2}^{*}=-0.4477$. By using the discomfort values derived with this optimal weights, the contribution of the kinematics related cost function can also be explained. Corresponding results are presented in Figures 8C,D.

Since human motor control is considered as a stochastic system and we do not know exactly how these factors are combined (e.g., linear or non-linear), the discomfort metric presented here is a proof-of-concept of the transition between different reaching tasks. Due to the absence of the description of the variance, the results contain noise, but the trade-off between the dynamics and the kinematics is still observable. This finding supports the idea to use a GPR model to describe the mapping from the initial and final joint angle configurations to the optimal weight vector.

### 5.2 Experiment for Hybrid Prediction Framework

In this subsection, an experiment designed to test the performance of the proposed hybrid online prediction framework is presented. The experiment is based on a simple pick-and-place task with one picking position and four targets. The accuracy of the ProMPs predictions as well as the updating process is analyzed here.

#### 5.2.1 Experimental Setup and Data Collection

**Figure 9:**
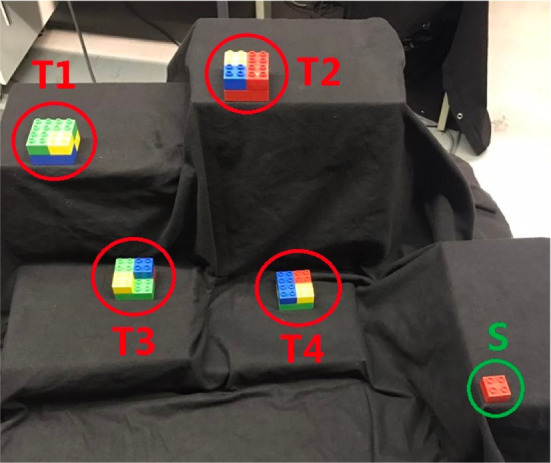
Experiment for the hybrid online prediction framework. S is the starting position and T1 to T4 are the four target regions. Each region consists of four possible placing positions as four corners of a square for the LEGO bricks.

As shown in Figure 9, the experiment is designed as a pick-and-place task with LEGO bricks. The picking position is fixed during the experiment, and four placing regions with different heights are selected as targets. Each region consists of four possible positions as four corners of a square for placing the bricks. Experiment includes 16 pick-and-place movements (4 targets × 4 times) per subject. Every subject is required to repeat the whole experiment ten times, thus in total 160 trajectories, 40 for each target, are recorded for one subject. We collected the data from five subjects and performed the analysis on those 800 trajectories. We neglect the hand and finger movements and only predict the position of the wrist joint.

#### 5.2.2 Results of the Hybrid Prediction Framework

Here, we present the corresponding results from the prediction experiment. First, the prediction accuracy of ProMPs is tested by looking into the distance error between the prediction and the observation. Then, the updating process for the GPR model is analyzed both to provide the evidence on the interpersonal variance, and also to demonstrate the ability of our hybrid prediction framework in describing this variance.

##### 5.2.2.1 Performance of the Predictions by ProMPs

We conduct an offline analysis to investigate the performance of the ProMPs-based predictions more in depth. After initialization, the ProMPs are utilized to generate predictions for the observations. For each observation, we use the first 30% of the observed points to rollout the prediction, and the distance error between the prediction and the observation is measured through DTW. After each prediction, the observation is used to update the ProMPs in order to learn the variance as well. For the next observation, the updated ProMPs is then used, and this updating process keeps running until the last observation.

**Figure 10:**
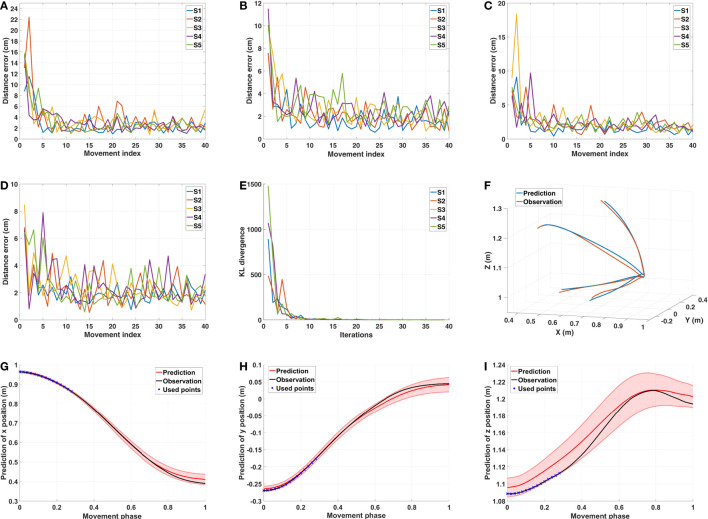
Results of predicting with the ProMPs. **(A–D)** Distance error between the observations and the ProMPs predictions of five subjects for target one to target four. The errors converge after several updates. **(E)** KL-divergence of comparing the updated distribution with the previous one for target one. It can be observed that after 10 iterations the value is quite small, which indicates that the distribution converges. **(F)** The ProMPs predictions and the observations in the last iteration of subject one for all four targets. **(G–I)** The ProMPs predictions in the last observation of subject one for target one. Each plot presented the mean and the variance of *x*,*y*, *z* positions in Cartesian space, respectively.

The distance errors for each subject and each target are presented in Figures 10A–D. The distance error is calculated between the prediction and the observation. Note that, this comparison is performed in Cartesian space, while during the initialization of the ProMPs, the trajectory generated from the composite model is a relative end-effector trajectory in arm model coordinate system (see Section 3.2.3). Since the relative end-effector trajectory ignores the shoulder translations and the torso movements, which are not avoidable in real reaching motions, and the model’s arm length is usually different than the actual arm length of the subject, the first prediction has large error. However, this initial error diminishes by later updates, and after several updates (around 5), the distance error becomes stable with a small value (around 2*–*4 cm for trajectory distance error averaged over the data points). In the end, as shown in Figure 10F, the predictions get closer to the observations for each subject.

During the prediction process, the variance is also learned by updating the ProMPs. We initialized the variance to a large value, and observe that after several updates the ProMPs converges to a stable distribution. Figure 10E shows the Kullback–Leibler (KL) divergence of comparing the updated ProMPs distribution with the previous one for target one. The results indicate that after around 10 iterations the distribution converges for each subject. An example of the learned distribution, which is defined by the mean values and the corresponding variances for each point in all dimensions, is presented in Figures 10G–I. Hence, the motor variability is captured by person-specific distribution in the ProMPs. Subsequently, the mean trajectory from the distribution is treated as the average behavior of that specific subject for the corresponding reaching task.

##### 5.2.2.2 Updating the GPR Model

**Figure 11:**
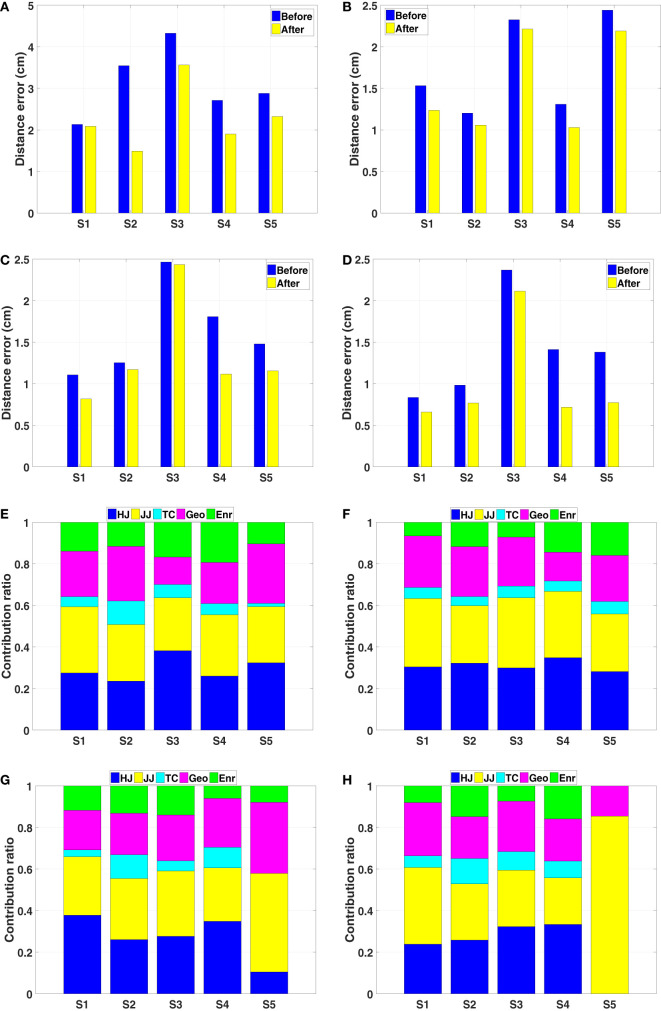
Results of the GPR model updating process. **(A–D)** The distance error between the optimal trajectories solved with respect to the initial weight vector and the updated weight vector for target one to target four, respectively. **(E–H)** The contribution of basic cost functions calculated from the mean trajectories of five subjects for target one to target four, respectively.

Due to the limited amount of available training data, the mapping represented by the GPR model is not accurate enough. Besides, because of the interpersonal variance, the error between the estimated weight vector and the actual one can be large in some cases. Thus, we need to update the GPR model through a separate updating process. To do this, we first extract the mean trajectory from the converged ProMPs learned from 40 observations, and then apply the same IOC calculation on this trajectory to get a new weight vector. This new weight vector is used to update the GPR model. Note that, since we also want to model the interpersonal variance, the GPR model is updated separately with respect to each subjects’ behavior. A comparison of the distance error between the observation and the optimal trajectories solved with the previous weight vector and the new weight vector is presented in Figures 11A–D. As we only want to look into the distance error caused by the weight vector, the trajectories compared here are the relative end-effector trajectories, which have less error due to ignoring the shoulder translations and the torso movements. The results indicate that the error diminishes after the update. After several updates on the GPR model, the interpersonal variance can be represented in each person-specific GPR model. We also observe that even for the same tasks the new weight vectors vary between different subjects (Figures 11E–H). This supports the existence of the interpersonal variance while emphasizing the importance of this updating process in our framework.

## 6 Discussion

Facilitating efficient and safe co-existence of humans and robots is a multifaceted challenge. In this paper, we focus on developing a human motion modeling and prediction framework that can be effectively used for robot control during dyadic interaction. One of the key insights of this work is that the interpersonal difference is not negligible regarding the contribution of cost functions. Even though motor variability was acknowledged in previous studies and some stochastic optimal control formulations were suggested as models for the motor control functionality of the CNS, the interpersonal variance has not been studied in such detail. The research presented in this work is a first step for combining model-based and probabilistic data-driven approaches in order to look into this topic, especially from the perspective of how this can be used for human-in-the-loop robot control. In essence, the hybrid framework enables personalized modeling and prediction of human motion behaviors, which can be integrated into robot control to provide personalized, safe, and efficient assistance to the human partner. However, there are still many aspects that need further investigation both for human motion modeling and its effective integration on robot control.

### 6.1 On the Human-in-the-Loop Robot Control and HRI

As robots have become ubiquitous in our daily lives, the goal is to provide safe yet natural interaction between human–robot dyads. To this end, novel robot control architectures which take into account human motion behavior are required. As robots are expected to adapt their motion behaviors with respect to their human counterparts, understanding how humans control and execute their motions is critical. The outcome of human motion modeling is twofold: on the one hand, the models learned can be used to predict human motions during interaction so that the robot can take proactive actions. On the other hand, such models enable building robot control architectures for realizing human-like motions to provide natural interaction. The proposed hybrid framework focuses on the former, and it also lays out the underlying control mechanism for human motor control while demonstrating the trade-off between kinematic and dynamic properties used for arm reaching control. Even though there were recent studies on transferring such optimal control formulations learned from human motion data to robot control (e.g., locomotion (Mombaur et al., 2010), reaching motion (Albrecht et al., 2011)), our findings would enhance such methods by building adaptive control methods to achieve a similar trade-off as human motor control seems to utilize.

The model-based optimal control formulation can further be utilized for other HRI settings, e.g., in physical HRI to provide the required assistance by the robot to the human partner in order to reduce the effort spent by the human which can be detected from the increase in dynamics related costs contribution. In addition, the trade-off analysis can be extended to understand how reciprocal influence of partners’ movement affect the cost distribution, which in turn help us construct suitable control and motion planning strategies for the robot to provide optimal assistance constrained on similar cost distributions.

As humans collaborate with each other naturally and safely in close proximity, we hypothesized that one crucial requirement for dyads is to be able to estimate the collaborating partner’s motions. In that regard, it is also essential for a robot to predict the motion of human partners. This prediction needs to be efficient (online-capable) in order to choose actions proactively, and to (re-)plan the motion in a way to realize a collision-free trajectory while still achieving the task. The proposed hybrid framework enables such an efficient prediction as well as an update on the cost combination per person. The ProMP-based human motion prediction component of this work has already been integrated into a stochastic trajectory optimization framework (Oguz et al., 2017). The efficiency of our motion prediction enables the robot to re-optimize its motion frequently at short intervals while considering the predicted human motion distribution as a dynamic obstacle to avoid. Hence, any changes in the expected movement can still be taken into account to achieve a responsive and safe interaction. Furthermore, since our hybrid architecture also updates personal motion models during interaction, the effect of robot movement on human partner’s motion can still be captured, which is expected to increase the accuracy of predictions during the course of the interaction.

In that regard, Interaction Primitives (IPs) (Amor et al., 2014) and its extension Environment-adaptive IPs (EaIPs) (Cui et al., 2016) also provide a data-driven approach to predict a human partner’s movement and then to plan the robot motion accordingly. As ProMP formulation already builds on the idea of learning a distribution over some demonstrated trajectories, it can also be extended to account for the coupling between two agents by learning a distribution over two persons’ trajectories executed during a joint interaction task. Similarly, learning a joint distribution including the environment-related features would be a feasible improvement. The learned human motion models can still be fed to the IOC formulation to extract the optimal cost distributions that best describes those interactive movement behaviors. The reciprocal influence of partners on their individual cost utilization poses an interesting research question that can be analyzed from the IOC perspective. Our modular hybrid framework also allows integration of any movement representation that can effectively predict human movement behaviors. In that regard, the IOC formulation can easily be integrated with (Ea)IPs to model, understand, and predict human interaction behaviors.

Finally, one critical issue has to be noted. Since those formulations only rely on data-driven formulations, there is no guarantee on a safe and effective motion generation for the robot, especially in close proximity interaction scenarios. However, our approach has the potential to utilize underlying cost function distributions learned from human movement behaviors for robot motion generation, which can then be combined with a learning approach to achieve a generalized safe policy. In that regard, we can combine the reachability analysis (Akametalu et al., 2014) with our model-based optimal control formulation to ensure the safety when the robot is planning its interaction movement. In essence, by the reachability analysis, the states that lead to an unsafe situation will be eliminated, and the learning process is performed within the safe region (Fisac et al., 2017). This analysis and the required computations are based on the dynamical model of the system and may not be feasible with the purely data-driven approaches, such as IPs.

### 6.2 Limitations

The IOC framework enables the identification of combination of basic cost functions in 3D reaching tasks. The results suggest a trade-off between the dynamics and kinematics related cost functions. With a proper definition of the system model and a set of reasonable cost functions, the IOC framework can be generalized to other problems, e.g., locomotion planning (Mombaur et al., 2010), car driving (Kraus et al., 2010). However, there are several limitations of the IOC framework, one of which is the complexity of finding the global minimum. Even though we tried to cover an extensive search range of the weight vector, the result is arguably still an approximation of the global minimum. Due to the complex non-linear formulation of the IOC framework, no efficient method has been proposed on addressing this problem yet. Second, the lack of the description of variance weakens the accuracy in terms of modeling the motion behavior. Since the IOC framework results in a deterministic solution, it cannot consider the interpersonal variance and the motor variability during the optimization. When we represent the trade-off between kinematics and dynamics related costs regarding the reaching tasks, the variance makes it hard to identify a clear relationship. Therefore, the discomfort metric we proposed is a proof-of-concept, and a deeper investigation is required to uncover how exactly the motion parameters affect the contribution of basic cost functions.

In the proposed hybrid prediction framework, we combine a model-based prediction method with a data-driven method. A GPR model is used to represent the mapping from the initial and final conditions to the optimal weight vector. However, due to the limited amount of data, the GPR model is not sufficient for representing the variance in motion behavior. It is also found to be effective only when the reaching motions are in the descriptive range of the training data. For prediction purpose, we use the trajectory obtained from the composite model to initialize the ProMPs. The reason we want to include this initialization phase other than directly using the ProMPs is that the subsequent updates on the composite models are much faster than solving the IOC problem from scratch for each person (e.g., 100 upper level optimization iterations take around 4 h vs. 15 iterations take around half an hour). It also allows to make the prediction immediately without extra data collection. Note that, because of the fact that the arm model ignores the shoulder translation and the torso movements, which are not avoidable in real reaching motions, the current initialization process still has some errors. If a full upper body model is considered in the IOC framework, this error could be minimized. However, this will immensely increase the computational load, hence this extension may not be feasible.

## 7 Conclusion

In this work, we investigate the underlying principles of human reaching motions and propose a hybrid framework to utilize our findings in motion prediction. To uncover the criteria of the reaching motion control, we implement an inverse optimal control framework to identify the contribution of basic cost functions which can best represent the human behaviors. The IOC results indicate a trade-off between the dynamics and kinematics related cost functions depending on the reaching tasks. Then to apply the composite cost function for predicting human motions, we combine the model-based optimal control formulation with the data-driven probabilistic movement primitives method. With this hybrid prediction framework, we learn the motor variability as well as the interpersonal variance at the same time. The demonstrated high accuracy and efficiency of this hybrid framework encourages its usage in HRI settings. For human-in-the-loop robot control, a high-level planner for the robot can exploit such a hybrid model to choose its next task, plan a collision-free motion trajectory, and as a result achieve safe, efficient, and natural dyadic interaction with the human partner.

## Ethics Statement

This study was carried out in accordance with the recommendations of Ethics Committee of Technical University of Munich (TUM) School of Medicine with written informed consent from all subjects. All subjects gave written informed consent in accordance with the Declaration of Helsinki. The protocol was approved by the Ethics Committee of TUM School of Medicine.

## Author Contributions

OO formulated and initiated the research. ZZ finalized the research and conducted the experiments. OO, ZZ, and DW wrote the paper.

## Conflict of Interest Statement

The authors declare that the research was conducted in the absence of any commercial or financial relationships that could be construed as a potential conflict of interest. The reviewer, MK, and handling Editor declared their shared affiliation.
